# Inter- Not Intraindividual Differences in sTWEAK Levels Predict Functional Deterioration and Mortality in Patients with Dilated Cardiomyopathy

**DOI:** 10.1155/2014/576482

**Published:** 2014-06-24

**Authors:** Kai-Uwe Jarr, Manfred Nelles, Hugo A. Katus, Emmanuel Chorianopoulos

**Affiliations:** Department of Cardiology, Angiology and Pulmonology, Heidelberg University Hospital, Im Neuenheimer Feld 410, 69120 Heidelberg, Germany

## Abstract

*Background*. TNF-like weak inducer of apoptosis (TWEAK) has been reported to predict mortality in patients with dilated cardiomyopathy. However, whether it can be used as a biomarker for disease monitoring or rather represents a risk factor for disease progression remains unclear. * Aim of the Study*. To evaluate the potential of sTWEAK as a biomarker in patients with dilated cardiomyopathy. * Results*. We conducted a serial study of sTWEAK levels in 78 patients with dilated cardiomyopathy. Soluble TWEAK levels predicted not only a combined mortality/heart transplantation endpoint after 4 years (*P* = 0.0001), but also the risk for clinical deterioration (*P* = 0.0001). Compared to NT-proBNP, sTWEAK remained relatively stable in individual patients on follow-up indicating that inter- rather than intraindividual differences in sTWEAK levels predicted outcome. Finally, neither did the scavenger receptor sCD163 correlate with sTWEAK levels nor did its determination add additional information on outcome in patients with dilated cardiomyopathy. * Conclusion*. Soluble TWEAK levels in patients with dilated cardiomyopathy may not be of value for disease monitoring but may represent a risk factor for disease progression and death. Further research will be necessary to elucidate the exact role of sTWEAK as a potential modulator of immune response in the setting of dilated cardiomyopathy.

## 1. Introduction 

Tumor necrosis factor- (TNF-) like weak inducer of apoptosis (TWEAK, also named TNFsf 12) is a member of the TNF-family of cytokines with multifunctional properties [1]. TWEAK is expressed as a type II transmembrane protein and then released into the interstitium after proteolysis of an active soluble 18 kD fragment (sTWEAK). TWEAK mediates its signaling through binding to its receptor, FGF-inducible 14 kD protein (Fn14, also TNFrsf12a or TWEAKR) [2]. In addition, TWEAK has also been reported to bind another membrane protein, the scavenger receptor CD163, although this has been questioned by others [3]. Like sTWEAK, CD163 can be released and measured in blood samples as a soluble fragment (sCD163). Levels of sCD163 in the circulation have been related to sTWEAK levels in a number of diseases like peripheral artery disease, diabetes, chronic renal failure, or systemic sclerosis [4–8].

Recently, a role for sTWEAK in cardiac pathophysiology has been proposed [5, 6, 9–11]. Interestingly and in contrast to other cytokines, sTWEAK serum levels were found to be reduced in patients with chronic heart failure and dilated cardiomyopathy as well as some other chronic diseases with increased inflammatory activity [4–7]. We and others have also reported the potential value of sTWEAK levels for the prediction of all-cause mortality in patients with chronic stable heart failure or dilated cardiomyopathy [12, 13]. However, it is still unclear whether sTWEAK has the potential as a biomarker for disease monitoring in patients with heart failure similar to NT-proBNP. In addition, why a single determination of sTWEAK predicts long-term outcome after a couple of years in patients with dilated cardiomyopathy remains unresolved.

Therefore, we conducted a follow-up study in a cohort of stable patients with dilated cardiomyopathy to evaluate the usefulness of serial sTWEAK determinations and their intraindividual variation over time in relation to their symptoms. Patients were seen on a yearly basis in our outpatient clinic with serial determination of clinical, functional, and laboratory parameters with follow-up assessment after four years.

## 2. Methods

### 2.1. Study Population

A cohort of seventy-eight stable patients diagnosed with dilated cardiomyopathy were identified and followed on a yearly basis in the heart failure clinic of our university hospital that serves as a tertiary referral center in southern Germany. Eligible patients had to have the diagnosis of dilated cardiomyopathy, to be at least 18 years of age, and to have a reduced left ventricular systolic function with an ejection fraction of less than 50% on echocardiographic evaluation. All examinees enrolled in the present study had to have a stable clinical condition and medication for at least 1 month before inclusion. Patients with malignant or inflammatory diseases, acutely decompensated heart failure (NYHA class IV), a history of organ transplantation, or significant acute/chronic renal failure (serum creatinine > 2 mg/dL) were not included in our study. Coronary angiography had been performed in all patients. Patients with significant coronary artery disease, considered to be responsible for the reduced left ventricular function, were also excluded from our study. Blood samples of these patients were taken on 3 consecutive follow-up visits once a year in addition to clinical and functional evaluation of the patients including a 6-minute walk test (6MWT) and a peak oxygen consumption study (peak VO_2_max). Soluble TWEAK, sCD163, and NT-proBNP levels were determined in different aliquots of the same blood sample. All patients included in our study completed a four-year follow-up visit or a telephone call. All patients gave written consent.

### 2.2. sTWEAK and sCD163 Level Determination

Blood samples were drawn in all patients once a year during a follow-up visit on three consecutive occasions. Plasma samples were generated within 30 minutes of collection by centrifugation at 1000 ×g for 10 minutes at 4°C. To avoid repetitive freeze-and-thaw cycles, aliquots were generated, immediately frozen, and stored at −80°C until analysis. Plasma samples were taken as part of a multiple biomarker registry project in patients with dilated cardiomyopathy and heart failure, for which only serial plasma samples were available. Soluble TWEAK levels were determined with an ELISA by use of a commercially available kit tested for determination of human plasma samples (Bender Medsystems, Vienna, Austria) according to the manufacturer's instructions. Briefly, a 1 : 2 diluted test sample was incubated for 3 hours at room temperature in wells precoated with an anti-human sTWEAK antibody together with a biotin-conjugated anti-human TWEAK antibody which binds to human TWEAK captured by the first antibody. Streptavidin-HRP binds to the biotin conjugated anti-human TWEAK. Following incubation unbound biotin conjugated anti-human TWEAK and streptavidin-HRP is removed during a wash step, and substrate solution reactive with HRP is added to the wells and wells are incubated for approximately 10 to 20 minutes. A colored product is formed in proportion to the amount of soluble human TWEAK present in the sample. The reaction is terminated by addition of acid and absorbance is measured at 450 nm. A standard curve is prepared from 7 human TWEAK standard dilutions and human TWEAK sample concentration is determined. Absorbance was measured with an automatic ELISA reader (Tecan Spectra, Crailsheim, Germany). Human sTWEAK is detected with this kit at a threshold of 9.7 pg/mL. Intra-assay and interassay coefficients of variation were 7.9% and 9.2%, respectively, according to the manufacturer. All measurements were performed in duplicate by an investigator unaware of patients' characteristics and outcome.

Soluble CD163 levels were determined with the MacroCD163 ELISA assay (IQ Products, Trillium Diagnostics). Soluble CD163 was only determined once at the time point of study initiation from the same sample as the first sTWEAK value was determined. Briefly, a polyclonal antibody recognizing CD163 is immobilized on the surface of a microtiter plate. After incubation with the sample or the standard containing recombinant CD163, a second biotinylated monoclonal antibody recognizing CD163 is added. Detection of the latter is done by adding streptavidin-HRP. Using TMB (3,3′,5,5′-tetramethylbenzidine) as a substrate for HRP, the amount of CD163 in the sample can be determined. Intra-assay variability of the assay is 3–6%; interassay variability is 5–8%, when measuring duplicates according to the manufacturer. As with sTWEAK determination, all samples were measured in duplicate.

### 2.3. NT-proBNP Plasma Levels

NT-proBNP was measured from different aliquots of the same plasma sample. Measurements were performed at the clinical core laboratory of the University Hospital Heidelberg with an ELISA (Roche Diagnostics, Mannheim, Germany).

### 2.4. Statistical Analysis

Data are presented as median (interquartile range) or count and percentages. For all continuous variables that were not normally distributed (tested by the Kolmogorov-Smirnov test), log transformation was performed. ANOVA was used for comparison of 3 or more different groups. The optimal plasma sTWEAK cutoff values to predict an adverse outcome in the present study population were calculated by a receiver operating characteristic (ROC) curve analysis. Estimates of the cumulative event rate were evaluated by the Kaplan-Meier method. Patients were compared according to the sTWEAK cutoff value with the use of log-rank test for the 4-year survival curves. Probability values < 0.05 were considered statistically significant. Statistical analyses were performed with Prism 5.0 (GraphPad Software, San Diego, California) and MedCalc 9.3.0.0 (MedCalc, Mariakerke, Belgium) software.

## 3. Results

### 3.1. Patient Characteristics

**Figure 1:**
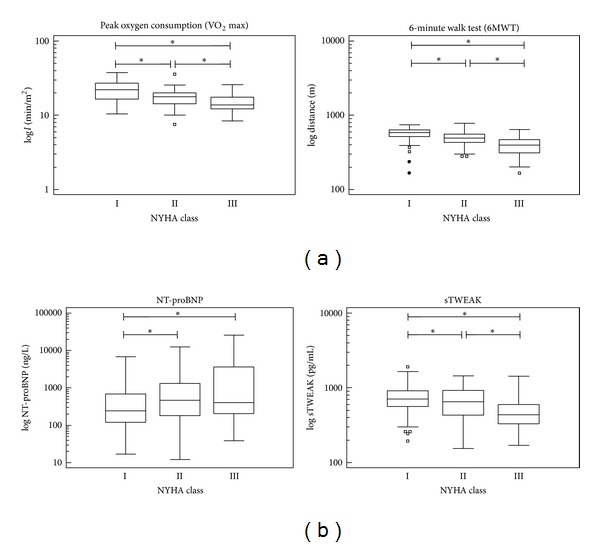
In our patient cohort of stable patients with dilated cardiomyopathy, reported functional NYHA class correlated well with functional testing. With increasing NYHA class, peak oxygen consumption (VO_2_max) and walking distance in the 6-minute walk test (6 MWT) progressively declined (a), whereas NT-proBNP levels increased (b). In contrast, sTWEAK levels significantly declined with increasing NYHA class (b). (Box-Whisker-Plots; *P < 0.05).

We serially analyzed a cohort of 78 patients diagnosed with dilated cardiomyopathy and evaluated sTWEAK levels, NT-proBNP levels, and functional parameters on a yearly outpatient basis in our heart failure clinic. The median ejection fraction (EF) of our patient cohort was 29% (interquartile range (IR) of 20 to 48%) and the median left ventricular end-diastolic diameter (LVEDD) was 60 mm with an interquartile range of 52 to 74 mm at the time point of study initiation. Median age of our patients was 55 years (IR: 43 to 64 years). Median of sTWEAK at study start was 603 pg/mL (IR: 423 to 839 pg/mL) and that of NT-proBNP was 482 ng/L (IR: 211 to 1505). Twenty-eight patients (35.9%) had atrial fibrillation, but only 4 (5.1%) developed thromboembolic events during follow-up. Overall NYHA class of the patient cohort was inversely related to peak oxygen consumption (VO_2_max) and walking distance during a 6MWT (peak oxygen consumption (VO_2_max): 22.0 (IR: 16.6 to 27.2) versus 17.7 (IR: 14.3 to 20.0) versus 13.7 (IR: 12.2 to 17.6); 6MWT: 587 (IR: 518 to 638) versus 492 (IR: 430 to 559) versus 395 (IR: 311 to 467); NYHA class I versus II versus III each; *P* < 0.05; Figure 1(a)). Moreover, NT-proBNP levels significantly increased with each NYHA class, whereas sTWEAK levels decreased (Figure 1(b)). Characteristics of our patient cohort are summarized in Table 1.

### 3.2. Baseline sTWEAK Predicts Mortality/Heart Transplantation and Functional Deterioration in Patients with Dilated Cardiomyopathy

**Figure 2:**
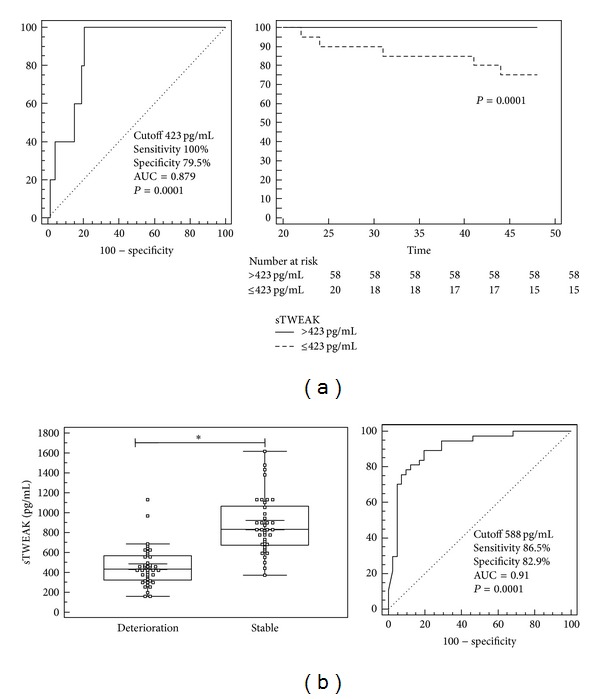
Soluble TWEAK predicts a progressive course of the disease in patients with dilated cardiomyopathy. (a) Receiver operating curve (ROC) analysis of baseline sTWEAK levels in patients with dilated cardiomyopathy revealed a cutoff value of 423 pg/mL for the prediction of death or heart transplantation on serial follow-up. Kaplan-Meier curves differed significantly between patients with a sTWEAK level below 423 pg/mL and those with an above level. (b) In those patients with significant deterioration during serial follow-up (*n* = 41), sTWEAK levels were already significantly reduced during the first visit. ROC analysis revealed a cutoff value of 588 pg/mL for the prediction of progressive symptoms (*P < 0.05).

Among our 78 patients, 4 deaths and 1 heart transplantation occurred within the 4-year follow-up period. Soluble TWEAK levels were lower in patients who died or were transplanted (372 pg/mL (239 to 405) versus 617 pg/mL (434 to 841); median (interquartile range); *P* = 0.0049). ROC curve analysis of baseline sTWEAK levels in our patient cohort revealed a cutoff value of 423 pg/mL for the prediction of death or heart transplantation (Figure 2(a)). Kaplan-Meier curve analysis demonstrated a significantly reduced survival in patients with a sTWEAK value below 423 pg/mL (Figure 2(a)).

We further compared those patients who reported later a significant deterioration of their functional capacity (≥1 NYHA class) over time to those with a stable condition on follow-up. In patients with progressive symptoms, sTWEAK levels were already significantly reduced at the first visit with a median of 432 pg/mL (IR: 322 to 568 pg/mL) (*n* = 41) compared to 833 pg/mL (IR: 674 to 1066 pg/mL) (*n* = 37) in stable patients (*P* < 0.0001) (Figure 2(b)). ROC curve analysis showed that a cutoff value of 588 pg/mL predicted a deterioration of at least 1 NYHA class during follow-up (Figure 2(b)).

### 3.3. Inter- Not Intraindividual Differences in sTWEAK Predict Subsequent Functional Deterioration and Mortality on Follow-Up

**Figure 3:**
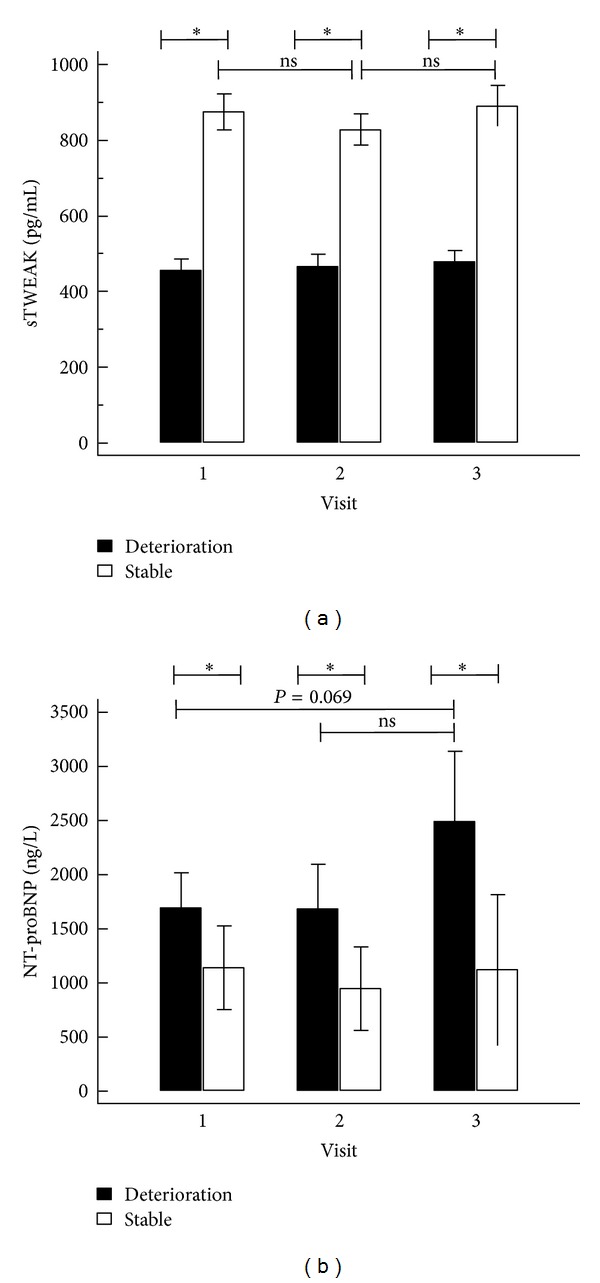
Interindividual differences in sTWEAK represent a risk factor for outcome in patients with dilated cardiomyopathy. To analyze the usefulness of sTWEAK as a biomarker in patients with dilated cardiomyopathy, we performed at least three serial sTWEAK measurements within all patients of our cohort. Interestingly, sTWEAK not only differed significantly between those patients with a progressive course of the disease and those with a stable course, but surprisingly remained very stable within individual patients with progressive deterioration, indicating that sTWEAK might represent a risk factor for disease progression rather than a biomarker for disease monitoring (*n* = 41; mean ± SEM; *P < 0.05).

Serial evaluation of sTWEAK levels allowed us to analyze whether sTWEAK showed intraindividual variations dependent on the actual clinical condition similar to the use of NT-proBNP. However, soluble TWEAK levels showed little variation in most patients throughout the follow-up period, no matter whether they developed progressive worsening of their clinical and functional parameters or not. Soluble TWEAK levels remained significantly lower in patients with deterioration compared to those with stable symptoms, indicating that interindividual rather than intraindividual differences predicted clinical deterioration (Figure 3). Similarly, sTWEAK levels correlated with maximum oxygen uptake during exercise (VO_2_max) and the results of the 6MWT, except for the first visit (6MWT) (first visit: VO_2_max: *r* = 0.3820; *P* = 0.0026; 6MWT: *P* = 0.1099; second visit: 6MWT: *r* = 0.3539; *P* = 0.0021; VO_2_max: *r* = 0.4693; *P* = 0.0001; third visit: 6MWT: *r* = 0.4086; *P* = 0.0002; VO_2_max: *r* = 0.4097; *P* = 0.0006). Similarly, when multivariate regression analysis was used to analyze an independent association between sTWEAK levels and VO_2_max over all visits, sTWEAK levels remained significantly associated (other variables associated with VO_2_max on univariate analysis: ejection fraction [*P* = 0.0258]; NT-proBNP [*P* = 0.0018]; retained variables in multivariate analysis: sTWEAK [*P* < 0.001]; NT-proBNP [*P* = 0.002]).

### 3.4. Soluble CD163 Levels Do Not Add Additional Prognostic Information to sTWEAK in Patients with Dilated Cardiomyopathy

Recently, the ratio between sCD163 and sTWEAK has been proposed to improve the predictive value of sTWEAK levels in patients with various clinical conditions [4–8]. We therefore analyzed sCD163 levels in our cohort of patients with dilated cardiomyopathy on their first visit and evaluated their relation to sTWEAK levels in the same sample. No significant association between sCD163 levels and sTWEAK levels was found (*P* = 0.7904). Compared to sTWEAK alone, ROC curve analysis of the sCD163/sTWEAK ratio in patients with dilated cardiomyopathy increased neither sensitivity nor specificity for the prediction of death/heart transplantation or clinical deterioration on follow-up (*P* = 0.650 for death/heart transplantation and *P* = 0.099 for clinical deterioration).

## 4. Discussion

Here, we report for the first time the results of a serial study of sTWEAK levels in a cohort of patients with dilated cardiomyopathy. Similar to previous reports, a single sTWEAK determination predicted long-term outcome in stable patients with dilated cardiomyopathy and reduced left ventricular function. In contrast to NT-proBNP, we found that sTWEAK showed little variation on serial determination within individual patients, independently of any clinical or functional deterioration. Thus, sTWEAK values differed significantly at baseline between individuals with subsequent progressive clinical deterioration and those with a stable clinical course. Moreover, sCD163 levels did not add additional information for outcome in our patient cohort. Finally, sCD163 did not correlate to the reduction of sTWEAK levels in patients with dilated cardiomyopathy.

The natural disease progression in patients with dilated cardiomyopathy can be quite variable despite similar echocardiographic and hemodynamic parameters. We and others have recently reported that reduced sTWEAK levels predicted long-term outcome in patients with dilated cardiomyopathy [12, 13]. However, how a single determination of sTWEAK could predict outcome and whether sTWEAK levels might be helpful for disease monitoring in these patients remained unclear. In addition, the role of sCD163, which has been related to sTWEAK levels in other diseases, was unknown for patients with dilated cardiomyopathy. Thus, our study provides new information not only about the usefulness of sTWEAK as a biomarker for patients with dilated cardiomyopathy, but also about the role of sCD163 in this setting.

Our study adds additional evidence that sTWEAK levels may not be useful for disease monitoring but may rather be related to an increased risk for disease progression in a subset of patients. This is of potential interest in the setting of dilated cardiomyopathy, since sTWEAK has also been related to several autoimmune disorders and vice versa autoimmunity has been suggested to play a role in the pathogenesis of patients with dilated cardiomyopathy. For example, autoantibodies against several molecular targets in the heart and additional features of immune activation like cytokine activation and intramyocardial inflammation are detectable even in apparently healthy relatives of patients with dilated cardiomyopathy [14–16]. Thus, decreased sTWEAK levels detected in the circulation of patients with dilated cardiomyopathy might represent a risk factor for a progressive course of the disease.

Whether sTWEAK plays a role in the pathogenesis of dilated cardiomyopathy or whether the association between reduced levels and outcome represents just an epiphenomenon of changes in the regulation of the immune system remains to be determined. However, recent experimental data suggest that sTWEAK directly influences cardiac pathology. Jain et al. have reported the occurrence of dilated cardiomyopathy in transgenic animals overexpressing TWEAK [17]. In addition, upregulation of Fn14 in stressed myocardium can be found in experimental models of heart failure [18, 19]. Increased clearance of sTWEAK due to chronic Fn14 upregulation in the heart may contribute to the reduced levels of sTWEAK found in chronic heart failure. However, sFn14 levels in serum or plasma were not determined in our patient cohort, mainly because their role for sTWEAK levels measured in the circulation has not been studied very well so far.

In contrast to sFn14, the ratio between sTWEAK and sCD163 has been described to be of value in other clinical settings, although a significant interaction between both molecules has been questioned by others [4–6, 20, 21]. Our data suggest that sCD163 is less likely to be responsible for the reduction of sTWEAK in patients with dilated cardiomyopathy. Moreover, the ratio between sCD163 and sTWEAK did not increase sensitivity or specificity for the prediction of an adverse outcome in patients with dilated cardiomyopathy. This is also in contrast to previous reports [4, 22].

Absolute values of sTWEAK levels in healthy control patients have been reported in several studies using different detection assays in the past reviewed in [23]. Usually reported serum levels of sTWEAK in a healthy Caucasian population range between 300 and 450 pg/mL, if measured with the assay we used in this study. Although we did not include a control cohort in our study, the median of sTWEAK levels detected in our patients with dilated cardiomyopathy was slightly higher. One of the reasons for this finding might be the use of plasma instead of serum for serial evaluation of sTWEAK levels, which could have affected absolute levels of sTWEAK.

We cannot completely rule out all confounders; nevertheless, our relatively small study cohort consisted of a homogenous patient population: coronary angiography was used in all patients to exclude significant coronary artery disease, which might have affected sTWEAK measurements. All patients included had three consecutive visits with determination of sTWEAK levels and functional testing and all of them completed 4-year follow-up. Almost all patients were on *β*-blockers and angiotensin-converting enzyme inhibitors or angiotensin receptor blockers and, therefore, on optimized therapy. All patients had to be clinically stable; patients with acute decompensation were not included. Patients with a history of malignant disease or a chronic inflammatory disease were also not included in our study.

In summary, our study is the first to provide data on serial sTWEAK determination in combination with serial clinical and functional assessment to evaluate the potential of sTWEAK as a biomarker in the setting of dilated cardiomyopathy. As a result, we found that the reduction of sTWEAK levels in patients with dilated cardiomyopathy may not be of value for disease monitoring but may represent a risk factor for disease progression and death. Further research will be necessary to elucidate the exact role of sTWEAK as a potential modulator of immune response especially in the setting of dilated cardiomyopathy.

## Figures and Tables

**Table 1:** Characteristics of the study cohort with dilated cardiomyopathy (*n* = 78).

Age (years)	55 (43–64)
Gender (m/f)	56/22
Ejection fraction in %	29.5 (20–45)
Left ventricular end-diastolic diameter (in mm)	60 (52–66)
6-minute walk test (distance in m)	503 (417–596)
Peak oxygen consumption (L/min/kg)	17.6 (14.5–23)
NYHA class	
Class I	36 (46.1%)
Class II	37 (47.4%)
Class III	5 (6.4%)
Class IV	0 (0%)
Medication	
*β*-Blockers	74 (94.9%)
ACEI/AT antagonists	77 (98.7%)
Aldosterone antagonists	41 (52.6%)
Diuretics	53 (67.9%)
Glycosides	23 (29.5%)
Diabetes	6 (7.7%)
Creatinine (mg/dL)	0.93 (0.8–1.2)
sTWEAK in pg/mL	603 (423–839)
sCD163 in ng/mL	2554 (1990–3479)
NT-proBNP in ng/L	482 (211–1505)
Death/heart transplantation on 4-year follow-up	4/1
Functional deterioration (≥1 NYHA class)	41 (52.6%)
Functional deterioration (≥2 NYHA classes)	11 (14.1%)

Values given as median (IR), number, or number (%).
